# FAIRifizierung von Real World Data für die Gesundheitsforschung: Ein Petitum für modernes Record Linkage

**DOI:** 10.1007/s11553-022-00973-x

**Published:** 2022-09-28

**Authors:** Iris Pigeot, Timm Intemann, Bianca Kollhorst, Ulrich Sax, Wolfgang Ahrens

**Affiliations:** 1 Leibniz-Institut für Präventionsforschung und Epidemiologie – BIPS, Achterstr. 30, 28359 Bremen, Deutschland; 2 Fachbereich Mathematik und Informatik, Universität Bremen, Bremen, Deutschland; 3 Fachbereich Medizininformatik, Universitätsmedizin Göttingen, Göttingen, Deutschland

**Keywords:** Datenschutz, FAIR-Prinzipien, Nationale Forschungsdateninfrastruktur, Unique Identifier, Verknüpfung personenbezogener Gesundheitsdaten, Data protection, FAIR principles, Linkage of person-related health data, National Research Data Infrastructure, Unique identifier

## Abstract

**Hintergrund:**

Die Bereitstellung von Real-World-Daten im Sinne der FAIR-Prinzipien ist die Voraussetzung einer effizienten Ausschöpfung des Potenzials von Gesundheitsdaten für Prävention und Versorgung.

**Ziel der Arbeit:**

Möglichkeiten und Limitationen der Nachnutzung und Verknüpfung von Gesundheitsdaten in Deutschland werden dargestellt.

**Material und Methoden:**

Es werden Initiativen zur Schaffung einer verbesserten Forschungsdateninfrastruktur vorgestellt und an einem Beispiel die Einschränkungen illustriert, die das Record Linkage personenbezogener Gesundheitsdaten behindern.

**Ergebnisse:**

In der Regel erfüllen Gesundheitsdaten in Deutschland nicht die Anforderungen der FAIR-Prinzipien. Ihre Auffindbarkeit scheitert bereits daran, dass entweder keine Metadaten zur Verfügung stehen oder diese nicht standardisiert in suchbare Repositorien eingestellt werden. Die Verknüpfung von personenbezogenen Gesundheitsdaten ist durch restriktive Datenschutzbestimmungen und das Fehlen eines sog. Unique Identifiers extrem eingeschränkt. Datenschutzkonforme Lösungen für die Verknüpfung von Gesundheitsdaten, die in europäischen Nachbarländern erfolgreich praktiziert werden, könnten hier als Vorbild dienen.

**Schlussfolgerung:**

Die Schaffung einer Nationalen Forschungsdateninfrastruktur (NFDI), insbesondere für personenbezogene Gesundheitsdaten (NFDI4Health), ist nur mit erheblichen Anstrengungen und Gesetzesänderungen realisierbar. Bereits vorliegende Strukturen und Standards, wie sie z. B. durch die Medizininformatik-Initiative und das Netzwerk Universitätsmedizin geschaffen wurden, sowie internationale Initiativen wie z. B. die European Open Science Cloud müssen dabei berücksichtigt werden.

## Abstract (en)

**Background:**

The provision of real-world data according to the FAIR principles is prerequisite for an efficient exploitation of the potential of health data for prevention and care.

**Objectives:**

To discuss the opportunities and limitations of reuse and record linkage of health data in Germany.

**Materials and methods:**

Initiatives to establish an improved research data infrastructure are presented and the limitations that hinder record linkage of personal health data are illustrated using an example.

**Results:**

In general, health data in Germany do not meet the requirements of the FAIR principles. Their findability already fails because either no metadata are available or they are not posted in searchable repositories in a standardized way. Record linkage of personal health data is extremely limited by restrictive data protection regulations and the lack of a so-called unique identifier. Privacy-compliant solutions for linking health data, which are successfully practiced in neighboring European countries, could serve as a model here.

**Conclusions:**

The establishment of a National Research Data Infrastructure (NFDI), especially for personal health data (NFDI4Health), can only be realized with considerable efforts and legislative changes. Already existing structures and standards that have been for instance developed by the Medical Informatics Initiative and the Netzwerk Universitätsmedizin (English: University Medicine Network), and international initiatives such as the European Open Science Cloud should be taken into consideration.

## Kurze Hinführung zum Thema

Zwar sind systematisch erhobene Forschungsdaten (sog. Primärdaten) die wesentliche Grundlage für wissenschaftliche Erkenntnis im Gesundheitsbereich, aber auch Daten, die z. B. routinemäßig zur Gesundheitsberichterstattung oder zu administrativen Zwecken erhoben werden (sog. Sekundärdaten), bieten ein großes, bislang nicht ausgeschöpftes Potenzial für die Erkenntnisgewinnung. Hier bedarf es einer Verbesserung der Möglichkeiten zu ihrer Nachnutzung („Data Sharing“) und Verknüpfung („Record Linkage“), um auf Basis einer optimierten Datenlage evidenzbasierte Entscheidungen im Gesundheitswesen zum Wohl der Bevölkerung treffen zu können.

## Hintergrund

Qualitätsgesicherte Forschungsdaten sind die wesentliche Grundlage für wissenschaftliche Erkenntnis in nahezu allen Disziplinen oder wie es Kiran Bhageshpur im Economist 2017 formulierte: „The world’s most valuable resource is no longer oil, but data“ [33]. Dabei kann in der Regel im Rahmen einzelner, thematisch eng begrenzter und zeitlich befristeter Forschungsprojekte das Potenzial der dafür gewonnenen Forschungsdaten nicht voll ausgeschöpft werden. Oft bieten diese Daten die Möglichkeit, weitere, zum Zeitpunkt der Erstellung noch nicht absehbare oder im jeweiligen Projekt nicht beantwortbare Fragestellungen zu bearbeiten, z. B. indem Daten verschiedener Projekte zusammengeführt werden, um den Stichprobenumfang zu vergrößern oder die Datentiefe zu erhöhen. Neben den spezifisch für ein Projekt erhobenen Daten werden in vielen Bereichen, z. B. zu administrativen Zwecken, weitere Daten erfasst, deren Potenzial zur Beantwortung von Forschungsfragen oft nicht erkannt wird und somit bei weitem nicht ausgeschöpft ist.

Um das gesamte Potenzial im Sinne einer effizienten Ressourcennutzung und zum Wohle der Gemeinschaft nutzen zu können, sollte die Forderung der Organisation für wirtschaftliche Zusammenarbeit und Entwicklung (OECD) von 2007, einen einfachen Zugang zu Forschungsdaten für die gesamte wissenschaftliche Community (Data Sharing) zu ermöglichen, auch auf andere, relevante Daten ausgeweitet werden. Dazu müssen für die Forschung relevante Daten entsprechend den sog. FAIR-Prinzipien [35] aufbereitet werden, d. h. sie müssen für Dritte jederzeit auffindbar („Findable“), zugänglich („Accessible“), interoperabel („Interoperable“) und wiederverwendbar („Reusable“) sein. Entsprechend dieser Forderungen wurde z. B. eine Initiative der Europäischen Kommission gestartet, die sog. European Open Science Cloud (EOSC; [7]), die europäischen Forschenden aber auch Unternehmen und Bürger:innen den Zugang zu wissenschaftlichen Daten erleichtern und geeignete Werkzeuge und Dienste zur Verfügung stellen soll.

Gerade im Gesundheitsbereich werden sowohl innerhalb als auch außerhalb des Forschungskontextes vielfältig Daten erzeugt, sei es zum Zweck der Gesundheitsberichtserstattung, bei der Durchführung epidemiologischer oder klinischer Studien, zu Abrechnungszwecken bei gesetzlichen oder privaten Krankenkassen, im Zuge der Erstellung und Pflege von Krankheitsregistern oder bei der Nutzung von Gesundheits-Apps und Wearables, um nur einige Beispiele zu nennen. Diese gesundheitsbezogenen Daten sind z. T. den sog. Real World Data zuzuordnen, die sich dadurch auszeichnen, dass sie außerhalb von klinischen Studien in heterogenen Populationen unter realen Bedingungen entstehen. Der Zugang zu und die Verknüpfung von personenbezogenen Datensätzen aus verschiedenen Quellen würden es nicht nur erlauben, ein umfassenderes Bild der Entwicklung von Krankheiten zu zeichnen und so bessere Maßnahmen zu deren Prävention zu entwickeln, sondern auch die therapeutischen Möglichkeiten sowie deren Chancen und Risiken im Einzelfall besser zu beurteilen. Natürlich muss die Nachnutzung solcher sensiblen Gesundheitsdaten hohen datenschutzrechtlichen und ethischen Standards genügen.

In Deutschland sind die Auffindbarkeit und die Nachnutzung von Daten noch sehr eingeschränkt, was die Forschung nicht nur im Gesundheitsbereich stark beeinträchtigt und den Wissenschaftsstandort Deutschland nachhaltig schwächt (s. dazu auch [32], in dem die Autor:innen 10 Empfehlungen für ein leistungsfähiges Forschungsdatenzentrum Gesundheit formulieren). In diesem Beitrag befassen wir uns mit den Schwierigkeiten des Datenzugangs sowie der Datenbereitstellung und -nachnutzung gemäß den FAIR-Prinzipien sowie mit den Möglichkeiten der Verarbeitung und Verknüpfung unterschiedlicher Datenquellen. Unser besonderes Augenmerk richtet sich dabei auf den Aufbau der Nationalen Forschungsdateninfrastruktur (NFDI), die unter Berücksichtigung internationaler Initiativen wie z. B. die EOSC zu erfolgen hat, und auf die Möglichkeiten des Record Linkage in Deutschland im Vergleich zum europäischen Ausland.

### NFDI4Health

In Deutschland wurde auf Empfehlung des Rats für Informationsinfrastrukturen [27] und nach einer entsprechenden Bund-Länder-Vereinbarung [6] im Jahr 2020 mit dem Aufbau einer NFDI [18] begonnen. Damit soll ein bundesweites, verteiltes und wachsendes Netzwerk zur systematischen Erschließung wissenschaftlicher Datenbestände sowie zur nachhaltigen Sicherung und Erhöhung der Zugänglichkeit dieser Datenbestände entstehen. Dieses soll später auch eine Vernetzung auf internationalem Niveau vorantreiben. Insgesamt werden nach drei aufeinanderfolgenden Ausschreibungsrunden bis zu 30 fachspezifische Konsortien mit einem Budget von bis zu 90 Mio. € pro Jahr gefördert.

Als eines der Konsortien, die bereits in der ersten Runde gefördert wurden, ist NFDI4Health [19] zum Aufbau einer NFDI für personenbezogene Gesundheitsdaten (Leitung: Juliane Fluck, Stellvertr.: Iris Pigeot) bereits im Oktober 2020 mit einer zunächst 5‑jährigen Laufzeit an den Start gegangen. NFDI4Health will Lösungen erarbeiten, die den besonderen Herausforderungen bei der Nachnutzung von personenbezogenen Gesundheitsdaten Rechnung tragen: Auch wenn es sich in den meisten Fällen um bereits strukturierte und qualitätsgesicherte Daten handelt, die in Forschungsprojekten gemäß standardisierten Studienprotokollen erhoben wurden, ergeben sich besondere Probleme dadurch, dass es sich dabei in der Regel um „lebende“ Datenkörper handelt. Die Personenbeziehbarkeit dieser sensiblen Daten und das Erfordernis zur ständigen Pflege, Aktualisierung und Fortschreibung longitudinaler Datenkörper führen dazu, dass ihre Nachnutzung durch Dritte nur für eingeschränkte Zwecke und nur unter Gewährleistung höchster Datenschutzanforderungen gestattet werden kann. Eine absolute Anonymisierung ist dabei aufgrund der üblicherweise in den Studien erfolgten tiefgehenden Phänotypisierung nicht möglich. Die Nutzungsmöglichkeiten der Daten sind darüber hinaus durch die jeweils erteilte informierte Einwilligung der Studienteilnehmer:innen beschränkt. Dazu kommt, dass ihre Auffindbarkeit trotz existierender Portale wie re3data.org [28] oder DataCite [5] beeinträchtigt ist und sich die Bereitstellung von Metadatenbeschreibungen noch nicht als Standard etablieren konnten. Auch die gemäß den FAIR-Prinzipien geforderte Interoperabilität zwischen verschiedenen Datenquellen ist noch nicht gegeben, da jede Institution ihre eigenen Standards entwickelt und anwendet.

NFDI4Health hat sich daher zum Ziel gesetzt, (1) die Auffindbarkeit von Gesundheitsdaten durch den Aufbau eines Central Search Hub zu verbessern, um die Publikation von Datensätzen und -beschreibungen zu unterstützen, Metadaten nach einheitlichen Standards zu dokumentieren und so zur Verbesserung der Interoperabilität beizutragen, (2) ein zentrales Datenzugangsportal („Central Data Access Point“) zu implementieren, (3) Prozesse aufzusetzen, die eine Nutzung nur im Einklang mit den gegebenen Einwilligungserklärungen und mit den geltenden Datenschutzrichtlinien gewährleisten, (4) Dienste weiterzuentwickeln, die einen geschützten Zugriff auf verteilt vorliegende Daten mittels Analysetools erlauben und (5) Dienste für eine dynamische und sichere Verknüpfung von Primär‑, Sekundär- und Registerdaten zu entwickeln. In all diese Aktivitäten sollen die Nutzer:innen eng eingebunden werden, um so eine große Akzeptanz zu erreichen und die Nachhaltigkeit der aufgesetzten Strukturen zu sichern.

## Schaffung einer verbesserten Wissensbasis durch Record Linkage

In Deutschland ist die Verknüpfung von personenbezogenen Sozial- und Gesundheitsdaten auf Basis personenidentifizierender Variablen mit sehr hohen datenschutzrechtlichen Hürden und einem hohen administrativen Aufwand verbunden: Zur Verknüpfung von Primärdaten ist die informierte Einwilligung der Studienteilnehmer:innen erforderlich. Ist die Einholung einer solchen Einwilligung nicht umsetzbar, wie z. B. bei der Verknüpfung von Sekundärdaten, muss die Einwilligung der Dateneigner und der jeweiligen Aufsichtsbehörden unter Vorlage eines genehmigungspflichtigen Datenschutzkonzepts eingeholt werden. Da zudem an den verschiedenen Stellen in der Regel nicht dieselben Pseudonyme verwendet werden bzw. verwendet werden dürfen und in Deutschland zurzeit noch kein Unique Identifier zum Record Linkage vorgesehen ist, ist in der Regel die Einrichtung von Vertrauensstellen erforderlich, die eine entsprechende (De‑)Pseudonymisierung vornehmen, um die verschiedenen Datensätze einer Person zu verknüpfen. Alternativ zur Verknüpfung der Daten über personenidentifizierende Variablen können verschiedene personenbezogene Datensätze auch über darin enthaltene individuelle Merkmale verknüpft werden. In Abhängigkeit von der Art, Anzahl und Genauigkeit der in den zu verknüpfenden Datensätzen hierfür zur Verfügung stehenden Merkmale kann eine hohe Anzahl an falschen Verknüpfungen auftreten. Dies hat dann erhebliche Einschränkungen bzgl. der Qualität der Ergebnisse zur Folge. So können beispielsweise Personen fälschlicherweise als Krebsfall klassifiziert werden, was zu verzerrten Analyseergebnissen führen und somit die Aussagekraft von Forschungsergebnissen erheblich einschränken kann.

Wie aufwändig die Verknüpfung zweier Datenquellen sein kann, zeigt das folgende Beispiel.

### Ergänzung fehlender Informationen in GKV-Daten durch Registerdaten

Daten Gesetzlicher Krankenkassen sind eine wertvolle Datenquelle sowohl für die Versorgungsforschung als auch für die pharmakoepidemiologische Forschung und zur Datenanreicherung von Beobachtungsstudien. Ihre Vorteile liegen in der großen Anzahl an Patient:innen, die zudem nicht-selektiert sind und für die über einen langen Beobachtungszeitraum Daten vorliegen. Somit werden Untersuchungen sehr seltener und sich spät manifestierender Ereignisse wie schwerwiegende Arzneimittelnebenwirkungen möglich. Zudem können vulnerable Bevölkerungsgruppen wie Kinder, Schwangere oder ältere Menschen in Studien berücksichtigt werden. Da die Daten allerdings primär zu Abrechnungszwecken erhoben und vorverarbeitet werden, ergeben sich Limitationen bei ihrer Nutzung in der Forschung. So besteht die Gefahr einer Über‑, Unter- oder Fehlkodierung von Diagnosen und Prozeduren/Operationen. Weiterhin fehlen aktuell Angaben zu Laborparametern, Lebensstilfaktoren, Tumorstadien oder Todesursachen. Fehlende Informationen könnten allerdings durch ein intelligentes Record Linkage ergänzt werden. So finden sich z. B. Daten zum Tumorstadium und dazu, ob ein:e Patient:in an einer Krebserkrankung verstorben ist, in den epidemiologischen und klinischen Krebsregistern der Bundesländer. Diese Registerdaten enthalten allerdings abgesehen von der Krebstherapie selbst keine Informationen zu Arzneimittelgebrauch oder Komorbiditäten. Damit lässt sich z. B. eine Studie, die eine Modifikation des Krebsrisikos durch Arzneimittelgebrauch untersucht, weder allein mit GKV-Daten noch allein mit Registerdaten sinnvoll durchführen.

**Abb. 1:**
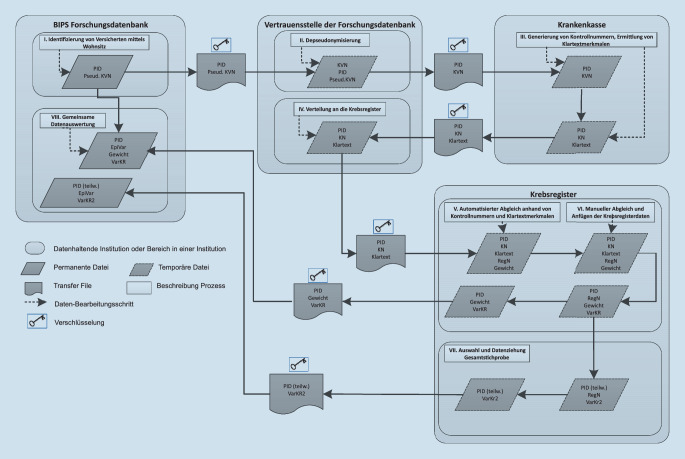
Datenfluss für die Durchführung beider Linkage-Verfahren (*PID* projektspezifische Identifikationsnummer, *Pseud. KVN* pseudonymisierte Krankenversicherungsnummer, *KVN* Krankenversicherungsnummer, *Klartext* Klartextmerkmale [Geburtsmonat und -jahr, Geschlecht, PLZ], *KN* Kontrollnummer, *RegN* registerinterne Nummer, *Gewicht* Übereinstimmungsgewicht, *VarKR* Variablen aus Krebsregister [Tumordiagnose, Datumsangaben zur Diagnose, TNM-Schlüssel und UICC-Stadium, Informationen zur Todesursache], *VarKR2* Variablen aus Krebsregister [Tumordiagnose, Datumsangaben zur Diagnose, TNM-Schlüssel und UICC-Stadium, Informationen zur Todesursache, GKZ, Geburtsjahr, Geschlecht], *EpiVar* Variablen aus der Forschungsdatenbank [Geburtsjahr, Geschlecht, Komobiditäten und Komedikation]). (Quelle: [22]. Die Abbildung ist von dieser Open-Access-Lizenz ausgenommen. Jede weitere Verwendung muss erneut und gesondert bei permission@thieme.de angefragt werden.)

In einer Studie gefördert durch die Deutsche Forschungsgemeinschaft wurde der Frage nachgegangen, inwieweit es möglich ist, die Daten der pharmakoepidemiologischen Forschungsdatenbank (GePaRD; [20]), die die Daten von vier GKVen enthält, mit den Daten epidemiologischer Krebsregister zu verknüpfen, um die Krankenversicherungsdaten um die fehlenden Informationen zum Tumorstadium zu ergänzen und die Validität der Tumordiagnosen abschätzen zu können. Da zudem zum Zeitpunkt der Durchführung des Projekts in beiden Datenquellen keine gemeinsame personenbezogene Identifikationsnummer zur Verknüpfung der Daten vorlag, die genutzt werden konnte, wurde in diesem Projekt zusätzlich der Frage nachgegangen, ob ein logistisch aufwändiges Verfahren mittels personenidentifizierender Merkmale, basierend auf in der Krebsregistrierung eingesetzten Kontrollnummern (kurz: direktes Linkage), durch ein weniger aufwändiges Verfahren (kurz: indirektes Linkage), basierend auf Merkmalen, die in beiden Datenquellen bereits vorhanden sind (Geburtsjahr [da das taggenaue Geburtsdatum aus Datenschutzgründen nicht in GePaRD enthalten ist], Geschlecht, Wohnort, basierend auf den ersten 5 Ziffern des Amtlichen Gemeindeschlüssels, Krebsart [definiert als die erste aufgetretene ambulante oder stationäre Diagnose; Darmkrebs: ICD-10 GM C18-C21, Schilddrüsenkarzinom: ICD-10 GM C73], Datum der Tumordiagnose), ersetzt werden kann. Das Ziel der Linkage-Verfahren war die Verknüpfung beider Datenquellen auf Personenebene. Zur Umsetzung der Linkage-Verfahren musste bei den zuständigen Behörden sowohl für GePaRD als auch für jedes epidemiologische Krebsregister die Genehmigung für beide Linkage-Ansätze eingeholt werden. Dabei zeigten sich große Unterschiede zwischen den Bundesländern bzgl. der vorgeschriebenen Antragsverfahren und der Auslegung des Bundeskrebsregistergesetzes, die eine große Variationsbreite von vollständiger Genehmigung über Teilgenehmigung bis hin zur vollständigen Ablehnung des Antrags zur Folge hatte [21]. Der nötige Datenfluss, um datenschutzrechtliche Aspekte für die Durchführung beider Linkage-Verfahren adäquat zu berücksichtigen, ist in Abb. 1 dargestellt.

Konkret wurden für zwei Krebsarten die Sensitivität (Anteil korrekt verknüpfter Personen) und Spezifität (Anteil nicht korrekt verknüpfter Personen) des indirekten Record Linkage untersucht. Dabei ergab sich für die Sensitivität 72,8 % für Darmkrebs und 71,4 % für das Schilddrüsenkarzinom. Die Spezifität lag bei beiden Krebsentitäten über 99 %. Eine Analyse der direkt verlinkten Daten ergab, dass sich die Ausprägungen der für das indirekte Linkage herangezogenen Verknüpfungsvariablen in beiden Datenquellen unterschieden. Dies betraf v. a. die Variable Krebsart: Ein Großteil der beim indirekten Linkage fälschlicherweise nicht verknüpften Fälle (falsch-negative Fälle) hatte zwar eine Krebsdiagnose in GePaRD kodiert, war aber im Krebsregister nicht erfasst. Möglicherweise wurde bei diesen Personen dem Krebsregister ein nicht-invasiver Tumor gemeldet, obwohl in den Kassendaten ein invasiver Tumor kodiert war. Nicht-invasive Tumoren waren für die Forschungsfrage nicht von Interesse und wurden daher nicht berücksichtigt. Ein weiterer Grund waren fehlende oder unterschiedlichen Angaben des Wohnorts, die dadurch zustande kommen, dass der Wohnort in den beiden Datenquellen zu verschiedenen Zeitpunkten erfasst wurde. Während im Krebsregister der Wohnort am Tag der Meldung erfasst wird, ist in den Krankenkassendaten nur der aktuelle Wohnort der versicherten Person am Tag des Datenabzugs vorhanden. Da GePaRD aber bereits seit 2004 besteht, konnte in diesem Fall der Wohnort am Tag der Krebsdiagnose über mehrere Datenlieferungen approximiert werden. Sollte die versicherte Person allerdings zum Zeitpunkt der Krebsdiagnose in einem Bundesland gelebt haben, dessen Krebsregister nicht an diesem Projekt teilgenommen hat, so war es trotzdem nicht möglich, die Daten zu verknüpfen.

Aufgrund der niedrigen Sensitivität ist eine indirekte Verknüpfung von Krankenkassen- und Krebsregisterdaten unter Verwendung von Merkmalen, die in beiden Datenquellen vorhanden sind, nicht empfehlenswert. Vielmehr sollte eine Verknüpfung beider Datenquellen über eine eindeutige Identifikationsnummer erfolgen, mit der sich ein Linkage schnell und einheitlich umsetzen lässt. Für die Verknüpfung von Krankenkassen- und Krebsregisterdaten wurden 2021 mit dem Gesetz zur Zusammenführung von Krebsregisterdaten die rechtlichen Rahmenbedingungen geschaffen, dass in Zukunft die Krankenversichertennummer zum Linkage verwendet werden kann [2]. Zudem besteht die Option für die Einführung einer personenbezogenen Identifikationsnummer durch die forschungskompatible elektronische Patientenakte nach §§ 341–355 Sozialgesetzbuch V zur semantischen Interoperabilität. Andere Möglichkeiten zur Verknüpfung – unabhängig von einem Kontakt mit dem medizinischen Versorgungswesen – böten die eID aus dem digitalen Personalausweis [3] oder die steuerliche Identifikationsnummer.

### Record Linkage im europäischen Vergleich

Anders als in Deutschland sind in anderen europäischen Ländern die Möglichkeiten zur personenbezogenen Verknüpfung verschiedener Datenquellen und somit zur effizienten Nutzung von Gesundheitsdaten wesentlich besser [31]. Dies liegt zum einen an den rechtlichen Rahmenbedingungen, die trotz einer einheitlichen EU-Datenschutzgrundverordnung in einigen Ländern ein direktes Record Linkage etwa durch einen Unique Identifier erlauben und zum anderen an der langen Tradition bei Aufbau, Pflege und Nutzung von verschiedensten bevölkerungsbezogenen Registern und Gesundheitsdatenbanken. Diese decken die gesamte Bevölkerung ab und stehen der Wissenschaft für personenbezogene Verknüpfungen mit anderen Datenquellen zur Verfügung. Zu diesen Ländern gehören Dänemark, Finnland, Island, Schweden, Norwegen, England, Estland, Niederlande, Schottland und Wales [16, 25, 26, 29, 34, 36]. Beispielsweise gibt es in Wales > 40 und in Dänemark > 100 solcher Datenbanken. Dazu gehören u. a. zentrale Datenbanken für Diagnosen, Verschreibungen und Behandlungen sowie krebs- oder andere krankheitsbezogene Register.

Zum Record Linkage werden in diesen Ländern in der Regel eindeutige nationale (Patienten-)Identifikationsnummern (ID) verwendet [34]. Dadurch wird das Record Linkage vereinfacht und Fehler bei der Zuordnung werden minimiert. Eine weitere Stärke dieses Systems besteht darin, dass zentrale Plattformen eingerichtet wurden, die einen Überblick über zur Verfügung stehende Datenbanken und Register bieten, wie z. B. die Secure Anonymised Information Linkage Databank in Wales [29]. In diesen Datenbanken werden nicht nur Gesundheitsdaten, sondern auch personenbezogene Daten aus anderen Bereichen wie beispielsweise Melde‑, Bildungs- und Einkommensdaten erfasst, die für die Gesundheitsforschung relevante Informationen enthalten. Diese Plattformen stellen sicher, dass die Datenbanken leicht auffindbar und für die Forschung zugänglich sind. Dass eine solche systematische Erfassung von Registern in Deutschland fehlt, bemängelte bereits der Sachverständigenrat zur Begutachtung der Entwicklung im Gesundheitswesen [31]. In einigen Ländern, z. B. Dänemark [8], besteht zudem die Möglichkeit, aggregierte Daten aus den zur Verfügung stehenden Datenbanken online abzurufen.

Die oben erwähnten Plattformen informieren außerdem über Antragsverfahren, Kosten für die Datenbereitstellung und -verknüpfung, Ansprechpersonen, Datenschutzregeln und gesetzliche Grundlagen (s. z. B. Finnland; [10]). Zudem erfolgen Antragstellung und Bewilligung – anders als in Deutschland (s. vorheriger Abschnitt) – in der Regel zentral. Im Falle der Verknüpfung mit Primärdaten (also Daten, die für einen spezifischen Studienzweck erhoben wurden) sind in den oben genannten Ländern allerdings auch wie in Deutschland in der Regel zuvor informierte Einwilligungserklärungen („informed consent“) bei den Teilnehmenden einzuholen.

An den folgenden zwei Beispielen zur Verknüpfung und Analyse von Real-World-Daten lässt sich das herausragende Potenzial für die Gesundheitsforschung in Ländern mit umfassenden Record-Linkage-Möglichkeiten sehr gut illustrieren:

- In England konnte durch die Verknüpfung von Daten aus Hausarztpraxen, Testlaboren, Krankenhäusern und Mortalitätsregistern von knapp 4 Mio. Jugendlichen bereits im September 2021 der Nutzen der SARS-CoV-2-Impfung nachgewiesen werden [11].
- In Dänemark wurde auf den verknüpften Daten aus Melde‑, Bildungs‑, Einkommens‑, Verschreibungs‑, Patienten‑, Pflege- und Mortalitätsregistern von mehr als 1,3 Mio. über 55-Jährigen ein Tool entwickelt, um das Risiko der Pflegebedürftigkeit älterer Menschen einschätzen zu können und eine intelligentere Steuerung von Pflegeangeboten zu ermöglichen [37].

Darüber hinaus wird der hohe Bedarf der Wissenschaft an solchen Daten deutlich, wenn man bedenkt, dass allein in Finnland seit Dezember 2020 über 300 Genehmigungen für Record-Linkage-Projekte erteilt wurden [9]. Insgesamt lässt sich festhalten, dass in einer Reihe von europäischen Ländern erforderliche Strukturen für die erfolgreiche Nutzung von Gesundheitsdaten für die Forschung geschaffen wurden. Deutschland hat hier noch einen deutlichen Nachholbedarf, wobei die in den oben genannten Ländern etablierten Prozeduren als Vorbild dienen können.

## Diskussion und Zusammenfassung

Eine verbesserte Nutzung von in der medizinischen Versorgung anfallenden Gesundheitsdaten wird seit vielen Jahren in der biomedizinischen Verbundforschung angestrebt und im Rahmen der Förderung einer nationalen Forschungsdateninfrastruktur verstärkt vorangetrieben. Als Beispiel für eine solche Verbundforschung seien die 21 Kompetenznetze in der Medizin genannt, mit denen das Bundesministerium für Bildung und Forschung (BMBF) systematisch für verschiedenen Krankheitsbilder die Zusammenführung von Versorgungsdaten und neu erhobenen Studiendaten initiiert und gefördert hat. So konnte im Rahmen des Kompetenznetzes „Angeborene Herzfehler“ ein Register, gespeist aus Versorgungsdaten und neu erhobenen Studiendaten, u. a. zur Ermittlung der Prävalenz von angeborenen Herzfehlern aufgebaut werden [15, 30]. Allerdings sind diese Daten in der Regel schwer aufzufinden, ihre personenbezogene Verknüpfung ist aus Datenschutzgründen kompliziert – wenn nicht sogar unmöglich – und, falls eine Verknüpfung theoretisch möglich ist, ist die Umsetzung aufwändig und fehlerbehaftet, da diese Daten ohne einen Unique Identifier gespeichert sind. Eine wichtige Ausnahme bilden die Krebsregisterdaten, die sich zum einen dadurch auszeichnen, dass sie gewissermaßen per Gesetzesregelung vollständig sind, da man einer Speicherung im Gegensatz zu anderen Krankheitsregistern nicht widersprechen kann. Zum anderen sind sie gut auffindbar sowie standardisiert und strukturiert verfügbar. Zudem werden nach dem neuen Gesetz zur Zusammenführung von Krebsregisterdaten auch Konzepte zur Verknüpfung mit anderen Datenquellen entwickelt, so z. B. mit den Abrechnungsdaten der gesetzlichen Krankenkassen.

Derzeit werden wertvolle Gesundheitsdaten in Datensilos aufbewahrt, die anlassbezogen z. T. bereits vor 30 Jahren nach dem damaligen Stand der Technik bzw. Standardisierung aufgebaut wurden. Die siloübergreifende Zusammenführung solcher Daten wurde versucht, jedoch aufgrund der angesprochenen Widrigkeiten selten erfolgreich umgesetzt, wie das Beispiel im 3. Abschnitt illustriert. Selbst in der COVID-19-Pandemie wurde sehr deutlich, dass zwar faktisch alle Daten für die systematische Beurteilung von Krankheitsverläufen und Risiken dieser Erkrankung erhoben und elektronisch gespeichert wurden, diese Daten jedoch – im Gegensatz zu anderen europäischen Ländern – nicht im Sinne der FAIR-Prinzipien genutzt werden konnten.

Bereits 2016 beklagten Wilkinson et al. die schlechte Auffindbarkeit von Datenbeständen und formulierten den dringenden Bedarf für eine verbesserte Infrastruktur, um die Nachnutzung von Daten für wissenschaftliche Fragestellungen zu unterstützen. Zu diesem Zweck erarbeiteten sie die FAIR Guiding Principles für die Aufbereitung und Verwaltung von entsprechenden Datenbeständen [35]. Diese Leitlinien zur Verbesserung der Verfügbarkeit der Daten scheinen simpel, sind in der Umsetzung jedoch sehr komplex: Um die Auffindbarkeit zu verbessern, fordern Wilkinson et al., dass jeder Datensatz mit einer eindeutigen Kennung, einem sog. Persistent Identifier, versehen wird. Damit alleine sind die Datenbestände allerdings nur eindeutig gekennzeichnet. Um sie auch finden zu können („Findable“), werden jedoch entsprechende Dienste und Verzeichnisse benötigt, in denen Forschende die für sie relevanten Daten mit den jeweiligen Datenbeschreibungen suchen können. Die Zugänglichkeit („Accessible“) erfordert die Durchsuchbarkeit von (Meta-)Daten mittels standardisierter Protokolle. Die Nachnutzbarkeit („Reusable“) hängt wiederum bei jedem einzelnen Datenbestand davon ab, wie und in welchem Kontext und aufgrund welcher gesetzlichen Grundlage die Daten gesammelt worden sind und kann durch eine gegebene Einwilligung sowohl zeitlich als thematisch stark eingeschränkt sein. Zudem ist es in vielen Fällen nicht möglich, eine informierte Einwilligung einzuholen, so dass für die Forschung dringend neue Erlaubnistatbestände oder gesetzliche Ausnahmen definiert werden müssen. Die vierte Komponente der FAIR-Prinzipien, die Interoperabilität, erfordert einen hohen Grad an Standardisierung, erleichtert dadurch jedoch nicht nur die Nachnutzung von Daten, sondern auch deren mögliche Zusammenführung im Sinne eines Record Linkage. Dabei stellt das Fehlen eines Unique Identifiers eine große Hürde dar, Daten aus verschiedenen Quellen patienten- bzw. probandenbezogen zusammenzuführen.

Da die Nutzung von direkten Identifikationsdaten nur im unmittelbaren Behandlungszusammenhang möglich ist, müssen die Gesundheitsdaten beim Herauslösen aus dem Primärkontext pseudonymisiert werden. Bevor solche personenbezogenen Gesundheitsdaten zu Forschungszwecken miteinander verknüpft werden dürfen, muss typischerweise eine informierte Einwilligung der Betroffenen eingeholt werden [14, 24]. Technische Konzepte dafür liegen seit mehr als 25 Jahren vor [17, 23]. Dabei kommen je nach Datenquelle unterschiedliche Pseudonymisierungsmethoden zum Einsatz – von simplen Excel-Tabellen, in denen die personenidentifizierenden Merkmale einer laufenden Nummer zugeordnet werden, bis hin zu Pseudonymen, die mit aufwändigen Bloom-Filtern erstellt werden und für eine fehlertolerante Verknüpfung von Datenbeständen genutzt werden können [17]. Zurzeit ist ein White Paper der NFDI4Health in Arbeit, in dem die Überlegungen zur Verbesserung der Verknüpfung von Gesundheitsdaten zusammengefasst werden.

Im Rahmen der Medizininformatik-Initiative (MII; [13]) und des während der COVID-19-Pandemie geschaffenen Netzwerks Universitätsmedizin (NUM; [4]) wurden sowohl föderierte als auch zentralisierte Ansätze zur Zusammenführung und Nachnutzung von Versorgungsdaten konzipiert und sukzessive umgesetzt. Besonderes Augenmerk wurde neben der Auffindbarkeit und Interoperabilität der Daten durch den Kerndatensatz der MII [1] auf die Verfügbarkeit der Daten anhand des MII Data Sharing Framework [12] gelegt.

## Fazit für die Praxis

- In Deutschland ist die Umsetzung von Record-Linkage-Verfahren für die Verknüpfung von personenbezogenen Gesundheitsdaten sehr aufwändig.
- In anderen europäischen Ländern kann dafür auf etablierte Strukturen, inklusive einer nationalen Identifikationsnummer, zurückgegriffen werden.
- Derzeit besteht eine Chance für die Einführung einer solchen Identifikationsnummer durch die forschungskompatible elektronische Patientenakte nach §§ 341–355 Sozialgesetzbuch V zur semantischen Interoperabilität.
- Epidemiologische Studien benötigen einen Identifier, der unabhängig von einem Kontakt mit dem medizinischen Versorgungswesen vergeben wird, z. B. die eID aus dem digitalen Personalausweis oder die steuerliche Identifikationsnummer.

## References

[1] Ammon D, Bietenbeck A, Boeker M, Ganslandt T, Heckmann S, Heitmann K, Sax U, Schepers J, Semler SC, Thun S, Zautke A (2019) Der Kerndatensatz der Medizininformatik-Initiative – Interoperable Spezifikation am Beispiel der Laborbefunde mittels LOINC und FHIR. Forum Med Dokumentation Med Inform 21:113–117

[2] Bundesgesetzblatt (2021) Gesetz zur Zusammenführung von Krebsregisterdaten. http://www.bgbl.de/xaver/bgbl/start.xav?startbk=Bundesanzeiger_BGBl&jumpTo=bgbl121s3890.pdf. Zugegriffen: 22. Juni 2022

[3] Bundesministerium des Inneren und für Heimat (2020) eID-Server. https://www.personalausweisportal.de/Webs/PA/DE/wirtschaft/technik/eID-server/eid-server-node.html. Zugegriffen: 20. Apr. 2022

[4] Bundesministerium für Bildung und Forschung (2021) CODEX bündelt Daten für die Covid-19-Forschung. https://www.gesundheitsforschung-bmbf.de/de/codex-bundelt-daten-fur-die-covid-19-forschung-12743.php. Zugegriffen: 23. Nov. 2021

[5] DataCite (2022) https://datacite.org/. Zugegriffen: 9. Mai 2022

[6] Die Gemeinsame Wissenschaftskonferenz (2018) Bund-Länder-Vereinbarung zu Aufbau und Förderung einer Nationalen Forschungsdateninfrastruktur (NFDI) vom 26. November 2018

[7] European Open Science Cloud (2022) https://eosc-portal.eu/. Zugegriffen: 18. Juni 2022

[8] eSundhed (2022) https://www.esundhed.dk/. Zugegriffen: 31. Jan. 2022

[9] Findata (2022) https://findata.fi/en/. Zugegriffen: 3. Jan. 2022

[10] Findata (2022) Services and instructions. https://findata.fi/en/services-and-instructions/. Zugegriffen: 31. Jan. 2022

[11] Gurdasani D, Bhatt S, Costello A et al (2021) Vaccinating adolescents against SARS-CoV‑2 in England: a risk–benefit analysis. J R Soc Med 114(11):513–524 34723680 10.1177/01410768211052589 PMC8649477

[12] Hund H, Wettstein R, Heidt CM et al (2021) Executing distributed healthcare and research processes—the hiGHmed data sharing framework. Stud Health Technol Inform 278:126–133 34042885 10.3233/SHTI210060

[13] Knaup P, Deserno T, Prokosch H‑U et al (2018) Implementation of a national framework to promote health data sharing. Yearb Med Inform 27(01):302–304

[14] Kohlmayer F, Lautenschläger R, Prasser F (2019) Pseudonymization for research data collection: is the juice worth the squeeze? BMC Med Inform Decis Mak 19(1):178 31484555 10.1186/s12911-019-0905-x PMC6727563

[15] Kompetenznetz Angeborene Herzfehler (2022) https://www.kompetenznetz-ahf.de/. Zugegriffen: 23. Juni 2022

[16] Kuiper JG, Bakker M, Penning-van Beest FJA et al (2020) Existing data sources for clinical epidemiology: the PHARMO database network. Clin Epidemiol 12:415–422 32425609 10.2147/CLEP.S247575 PMC7196787

[17] March S, Andrich S, Drepper J et al (2019) Gute Praxis Datenlinkage (GPD) (Good Practice Data Linkage). Gesundheitswesen 81(08/09):636–650 31394579 10.1055/a-0962-9933

[18] Nationale Forschungsdateninfrastruktur (NFDI) e. V. (2022) Nationale Forschungsdateninfrastruktur. https://www.nfdi.de/. Zugegriffen: 20. Febr. 2022

[19] NFDI4Health (2022) Nationale Forschungsdateninfrastruktur für personenbezogene Gesundheitsdaten. https://www.nfdi4health.de/. Zugegriffen: 20. Febr. 2022

[20] Pigeot I, Ahrens W (2008) Establishment of a pharmacoepidemiological database in Germany: methodological potential, scientific value and practical limitations. Pharmacoepidemiol Drug Saf 17(3):215–223 18200610 10.1002/pds.1545

[21] Pigeot I, Bongaerts B, Eberle A et al (2022) Verknüpfung von Abrechnungsdaten gesetzlicher Krankenkassen mit Daten epidemiologischer Krebsregister: länderspezifische Möglichkeiten und Limitationen (Linkage of claims data with data from epidemiological cancer registries: possibilities and limitations in the German federal states). Bundesgesundheitsblatt Gesundheitsforschung Gesundheitsschutz 65(5):615–623 34940893 10.1007/s00103-021-03475-x PMC9064838

[22] Pigeot I, Kollhorst B, Didelez V (2021) Nutzung von Sekundärdaten für die pharmakoepidemiologische Forschung – machen wir das Beste draus! (Secondary Data for Pharmacoepidemiological Research—Making the Best of It!). Gesundheitswesen 83(S 02):S69–S76 34695869 10.1055/a-1633-3827

[23] Pommerening K, Miller M, Schmidtmann I et al (1996) Pseudonyms for cancer registries. Methods Inf Med 35(2):112–121 8755384

[24] Pommerening K, Reng M, Debold P et al (2005) Pseudonymisierung in der medizinischen Forschung – das generische TMF-Datenschutzkonzept. GMS Med Inform Biom Epidemiol 1(3):Doc17

[25] Pukkala E, Engholm G, Højsgaard Schmidt LK et al (2018) Nordic Cancer Registries—an overview of their procedures and data comparability. Acta Oncol 57(4):440–455 29226751 10.1080/0284186X.2017.1407039

[26] Rahu K, McKee M, Mägi M et al (2020) The fall and rise of cancer registration in Estonia: the dangers of overzealous application of data protection. Cancer Epidemiol 66:101708 32446217 10.1016/j.canep.2020.101708

[27] Rat für Informationsinfrastrukturen (RfII) (2016) Leistung aus Vielfalt. https://rfii.de/download/rfii-empfehlungen-2016/. Zugegriffen: 20. Febr. 2022

[28] re3data (2022) Registry of research data repositories. https://www.re3data.org/. Zugegriffen: 9. Mai 2022

[29] SAIL Databank (2022) The secure anonymised information linkage databank. https://saildatabank.com/. Zugegriffen: 31. Jan. 2022

[30] Schwedler G, Lindinger A, Lange PE et al (2011) Frequency and spectrum of congenital heart defects among live births in Germany: a study of the competence network for congenital heart defects. Clin Res Cardiol 100(12):1111–1117 21909849 10.1007/s00392-011-0355-7

[31] Gesundheit SVR (2021) Gutachten 2021: Digitalisierung für Gesundheit. https://www.svr-gesundheit.de/gutachten/gutachten-2021. Zugegriffen: 27. Jan. 2021

[32] Swart E, Gothe H, Hoffmann F et al (2021) Jetzt die Weichen stellen für ein leistungsfähiges Forschungsdatenzentrum Gesundheit. Gesundheitswesen 83(S02):S139–S141 34695868 10.1055/a-1537-9722

[33] The Economist (2017) The world’s most valuable resource is no longer oil, but data. https://www.economist.com/leaders/2017/05/06/the-worlds-most-valuable-resource-is-no-longer-oil-but-data. Zugegriffen: 20. Febr. 2022

[34] van Herk-Sukel MPP, Lemmens VEPP, van de Poll-Franse LV et al (2012) Record linkage for pharmacoepidemiological studies in cancer patients. Pharmacoepidemiol Drug Saf 21(1):94–103 21812067 10.1002/pds.2205

[35] Wilkinson MD, Dumontier M, Aalbersberg IJJ et al (2016) The FAIR Guiding Principles for scientific data management and stewardship. Sci Data 3:160018 26978244 10.1038/sdata.2016.18 PMC4792175

[36] Wood A, Denholm R, Hollings S et al (2021) Linked electronic health records for research on a nationwide cohort of more than 54 million people in England: data resource. BMJ 373:n826 33827854 10.1136/bmj.n826 PMC8413899

[37] Wright MN, Kusumastuti S, Mortensen LH et al (2021) Personalised need of care in an ageing society: The making of a prediction tool based on register data. Royal Stat Soc Ser A 184(4):1199–1219

